# Observation of directly interacting coherent two-level systems in an amorphous material

**DOI:** 10.1038/ncomms7182

**Published:** 2015-02-05

**Authors:** Jürgen Lisenfeld, Grigorij J. Grabovskij, Clemens Müller, Jared H. Cole, Georg Weiss, Alexey V. Ustinov

**Affiliations:** 1 Physikalisches Institut, Karlsruhe Institute of Technology, 76131 Karlsruhe, Germany; 2 Département de Physique, Université de Sherbrooke, Sherbrooke, Québec, Canada J1K 2R1; 3 ARC Centre of Excellence for Engineered Quantum Systems, School of Mathematics and Physics, University of Queensland, Brisbane, Queensland 4072, Australia; 4 Chemical and Quantum Physics, School of Applied Sciences, RMIT University, Melbourne 3001, Australia; 5 National University of Science and Technology MISIS, Leninsky prosp. 4, Moscow, 119049, Russia

## Abstract

Parasitic two-level tunnelling systems originating from structural material defects affect the functionality of various microfabricated devices by acting as a source of noise. In particular, superconducting quantum bits may be sensitive to even single defects when these reside in the tunnel barrier of the qubit’s Josephson junctions, and this can be exploited to observe and manipulate the quantum states of individual tunnelling systems. Here, we detect and fully characterize a system of two strongly interacting defects using a novel technique for high-resolution spectroscopy. Mutual defect coupling has been conjectured to explain various anomalies of glasses, and was recently suggested as the origin of low-frequency noise in superconducting devices. Our study provides conclusive evidence of defect interactions with full access to the individual constituents, demonstrating the potential of superconducting qubits for studying material defects. All our observations are consistent with the assumption that defects are generated by atomic tunnelling.

## Abstract (web summary)

Material defects create spurious two-level systems that are a source of noise in nanostructured devices. Lisenfeld *et al.* use a superconducting qubit to perform high-resolution defect spectroscopy, providing direct evidence of quantum coherent interaction between two defects.

## Graphical abstract

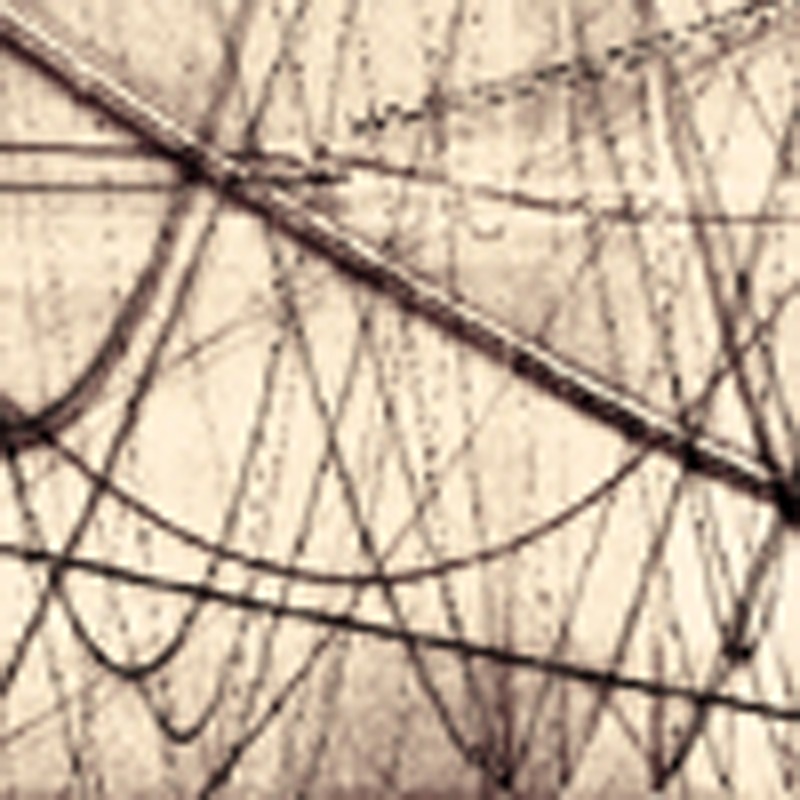

Superconducting quantum bits^1^ have recently achieved a breakthrough by demonstrating excellent gate fidelities and long coherence times in a fully scalable architecture^2^, placing the realization of an integrated quantum computing chip within reach. The solid-state approach, however, bears the burden that the material of the quantum device itself may host parasitic defects that give rise to two-level systems (TLS) acting as a sparse decohering bath.

First signatures of coherent TLS in phase qubits were found in spectroscopy data, where observed avoided level crossings manifest the defects’ two-level quantum character^3^,^4^. Often, these defects show longer coherence times than the qubit itself^5^, and thus might be useful as quantum memories^6^ and resources for quantum algorithms^7^,^8^. Phase qubits were used in several attempts to identify the physical origin of those TLS, for example, by obtaining statistics on frequencies and coupling strengths^9^, estimating their density^4^, measuring the temperature dependence of their coherence times^5^ or verifying theoretical models describing their origin^10^.

The possibility of a direct interaction between TLS has been invoked in the past to explain the line width broadening and spectral diffusion of ultrasonically excited ensembles of TLS in glasses^11^,^12^ as well as various other low-temperature properties of disordered solids^13^,^14^. TLS are furthermore a widely accepted model to explain noise in superconducting circuits, and mutual TLS coupling was recently suggested as the origin of the low-frequency noise observed in microwave resonators^15^.

Here, we report the first clear experimental evidence of two coherently interacting TLS residing in the tunnel barrier of a Josephson junction (JJ). The data are obtained with a new technique for high-resolution defect spectroscopy that exploits the tunability of TLS by mechanical strain and their strong coupling to a superconducting qubit. To characterize the coupled defect system in more detail, we build on this technique and perform coherent two-photon spectroscopy that directly reveals the TLS’ coupling strengths and independent parameters. Interpretation of the measurement based on atomic tunnelling systems fully accounts for all observations.

## Results

### Atomic tunnelling systems

To explain the microscopic origin of the TLS in superconducting electronics, several models^16^,^17^,^18^,^19^,^20^ have been proposed. However, all experimental results obtained so far, including the recent demonstration that the energy of the TLS is tunable by static mechanical strain^21^, are readily explained assuming that they originate from atomic tunnelling systems. As in the well-studied model describing the low-temperature thermal, dielectric and acoustic properties of disordered solids^22^,^23^,^24^, it is assumed that some atoms or small groups of atoms are able to tunnel between two energetically almost equivalent sites within the disordered oxide material of the device. These systems give rise to two-level excitations in a wide energy range of up to the order *E*≈*k*_*B*_·1 K or ≈*h*·20 GHz. In bulk disordered solids, TLS are found in large numbers but, in contrast to their counterparts present in superconducting qubits, cannot be addressed individually.

**Figure 1:**
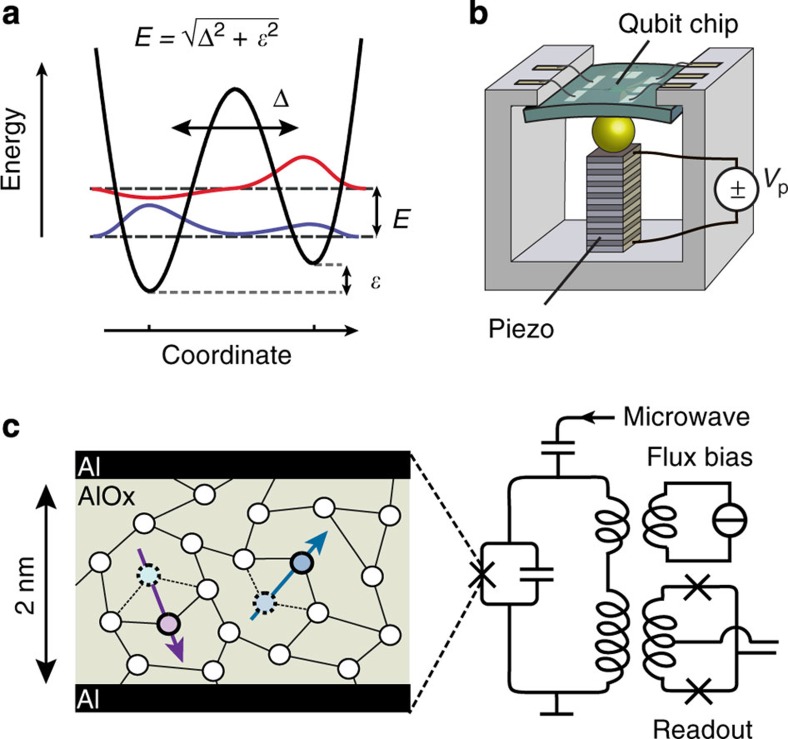
Using a superconducting qubit to access defects in JJs. (**a**) Illustration of the double-well potential for an atomic tunnelling system. Tunnelling energy Δ and asymmetry energy *ε* determine the level splitting *E*. (**b**) Sketch of the sample holder. To control the strain, the qubit chip is bent by applying a voltage *V*_p_ to the stacked piezo actuator. (**c**) Schematic of the phase qubit including manipulation and measurement circuitry. The JJ tunnel barrier is sketched as a disordered insulator hosting TLS defects, here pictured as atoms tunnelling between two metastable positions, with the arrows illustrating their electric dipole moment.

According to the tunnelling model, an atomic tunnelling system is described as a particle in a double-well potential as shown in Fig. 1a. The energies of the two wells differ by the asymmetry *ε* and the tunnelling amplitude between them is denoted as Δ, resulting in a level splitting of the two eigenstates given by 
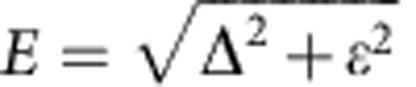
. Tunnelling systems couple to the environment predominantly by variation *δε* of their asymmetry energy with *δε* depending linearly on strain fields and, if the tunnelling entity moves a non-zero charge, as well on electric field—the latter serving as an apparent explanation for the observed coupling of the TLS to the qubit circuit. A variation *δ*Δ of the tunnelling amplitude induced by strain or external fields is generally believed to be negligible^12^,^24^. We have recently verified the linear strain dependence of *ε* and the corresponding hyperbolic variation of the energy splitting *E* by tracking individual TLS with a phase qubit while bending the chip circuit with a piezo actuator^21^. In our setup, sketched in Fig. 1b, an applied piezo voltage *V*_p_ results in variable strain fields in the order of 10^−6^/*V*.

### Defect spectroscopy

In this study, we detect and analyse TLS using a superconducting qubit. These devices rely on JJs as nonlinear circuit elements, which are realized as two superconducting films separated by a thin, insulating tunnelling barrier, consisting of a 2–3-nm-thick structurally disordered layer of aluminium oxide. A sketch of the employed phase qubit^25^ including measurement and manipulation circuitry is shown in Fig. 1c. The qubit’s level splitting and their population are controlled by externally applied flux bias and resonant microwave pulses, respectively, and a DC-SQUID is used for qubit readout.

**Figure 2:**
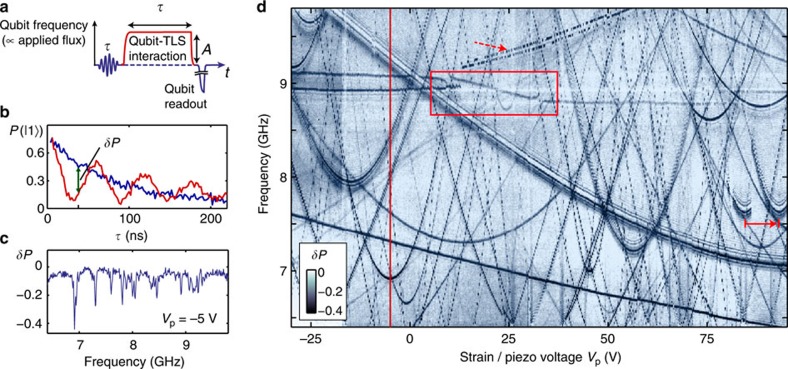
Defect spectroscopy. (**a**) Pulse sequence used to detect TLS via resonant interaction with the qubit. The qubit is excited by a *π*-pulse, tuned to varying probe frequencies using different flux-pulse amplitudes *A*, and its population is measured after an interaction time *τ*. (**b**) Qubit population probability, measured for two different probing frequencies in dependence of the interaction time *τ*. The blue curve shows purely exponential energy relaxation for the isolated qubit, the red curve displays oscillations owing to a strongly coupled and coherent TLS. We take the difference between the curves, measured at a fixed *τ* as indicated, for the defect signal *δP*. (**c**) Defect signal *δP* in dependence of the probing frequency for a fixed *τ*. Individual TLS appear as pronounced dips. (**d**) Strain dependence of TLS resonance frequencies, appearing as dark traces in *δP*, indicating a reduction in qubit population owing to its resonant coupling to a TLS. Mutual TLS interactions are observed as random switching of the TLS resonance frequency (arrows), avoided level crossings and non-hyperbolic traces (box). The cross-section at *V*_p_= −5 V (vertical line) is shown in **c**.

In order to trace the energies of individual TLS while applying strain to the qubit chip, we use a spectroscopy scheme based on the pulse protocol depicted in Fig. 2a. The qubit is first biased at a frequency far away from the intended spectroscopy region and excited by a resonant microwave *π*-pulse. Applying appropriate flux bias, it is then tuned to the probing frequency *f*_*h*_ where it resides for the holding time *τ*. If at this frequency the qubit is in resonance with a certain TLS, the excitation is shared between the systems^6^,^9^. This results in coherent oscillations that effectively swap the quantum states of the two systems at a frequency determined by their coupling strength as shown in Fig. 2b. A change *δP* in the qubit excitation probability, measured after the interaction time *τ*, thus reveals the presence of a TLS. We chose *τ* to be about half the qubit’s life and coherence times *T*_1_ and *T*_2_ (here, both ≈100 ns) to reach a compromise between the loss of signal due to qubit relaxation and the sensitivity needed for detecting also weakly coupled TLS. We repeat this procedure for a range of probing frequencies *f*_*h*_ and vary the mechanical strain applied to the sample.

By plotting the change in qubit population as a function of both applied strain and probing frequency, we obtain defect spectra like the one shown in Fig. 2d. Dark traces indicate the resonance frequencies of individual TLS, which are tuned by strain as expected for atomic tunnelling systems. Some TLS have a tunnelling energy Δ that falls within the frequency range accessible by the qubit (~6.5–10 GHz for our sample), while also their asymmetry energy *ε* is tuned through zero in the investigated strain range. Accordingly, for those TLS, we can clearly observe the hyperbolic strain dependence of their resonance frequencies around minima given by Δ/*h*. We note that the distribution of TLS resonances changes completely once the sample is warmed to room temperature; see Supplementary Fig. 1 for various examples. This can be explained by a modification of the atomic configuration changing the TLS environment locally, and also by an offset in the applied strain due to thermal dilatation of the sample fixture. As long as the temperature is kept below ~10–20 K, the properties of the majority of TLS remain constant over several months of measurements.

For TLS that are strongly coupled to the qubit, the chosen interaction time *τ* may exceed the duration of one swap operation. Since the latter also depends on the detuning, a fringe-like interference pattern occurs around the traces; see Supplementary Fig. 2. More details on this effect and other artefacts in such defect spectra are discussed in Supplementary Note 1.

### Mutually coupled TLS

In the defect spectroscopy example shown in Fig. 2d, two different effects of interactions between TLS can be identified: (i) the resonance frequency of certain TLS is observed to switch between two values in a random, telegraph-signal-like fashion, with a switching frequency that depends on the applied strain. This effect is explained by assuming that the observed TLS couples non-resonantly to an incoherent defect that fluctuates between its positions. (ii) Much less frequently observed are non-hyperbolic strain responses in the TLS resonance frequency as well as level splittings that are the typical signature of two resonantly coupled coherent quantum systems.

**Figure 3:**
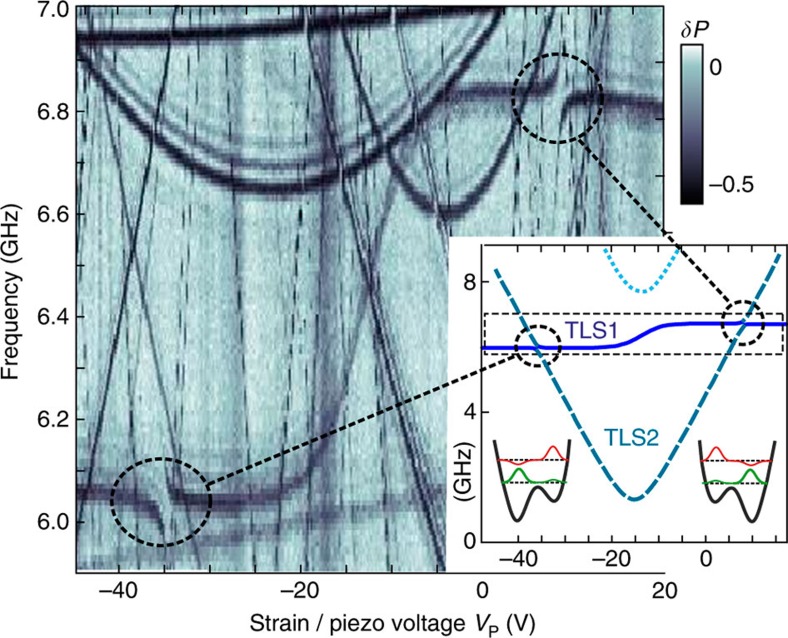
Spectroscopic signature of two mutually interacting TLS. The observed S-shaped feature with avoided level crossings (encircled) is characteristic for a TLS (TLS1) that is coherently coupled to a second defect (TLS2). The inset shows their spectrum calculated from theory, closely reproducing the data obtained between 6 and 7 GHz (dashed box). Here, TLS2 is strain-tuned through its symmetry point *ε*_2_=0 and changes the location of the ground state in its double-well potential (insets), while TLS1 is only weakly influenced by strain.

In the remainder of this article, we discuss a particularly clear manifestation of a system of two coupled TLS, whose defect spectroscopy signature revealed the S-shaped trace with avoided level crossings shown in Fig. 3. To explain this spectroscopic feature, we construct a model of two interacting defects denoted as ‘TLS1’ and ‘TLS2’ (see also inset to Fig. 3). Qualitatively speaking, we observe that TLS1 couples only weakly to the applied external strain so that the step-like increase of its resonance frequency is almost exclusively owing to its non-resonant coupling to TLS2. In the region shown, the double-well potential of TLS2 is strain-tuned through its symmetry point *ε*_2_(*V*_p_)=0 at *V*_p_≈ −14 *V* such that the probability density of its ground state is shifted gradually from one potential well to the other. The accompanied shift in atomic positions is mediated via internal strain or electric field to TLS1, which responds by modifying its energy splitting, here from 6 to 6.8 GHz. Eventually, the applied strain tunes TLS2 into resonance with TLS1 and their coherent interaction gives rise to the associated and observed level repulsions.

In the following, we outline our theory of the coupled defect system and show how it can be fully characterized by analysing strain-spectroscopic data. The Hamiltonian of a single TLS is written as:


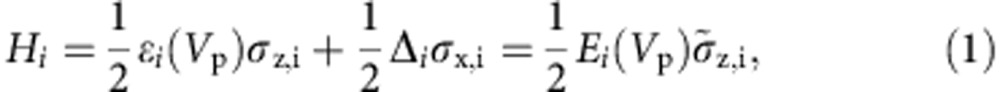


where Δ_*i*_ is the tunnelling energy and the asymmetry energy ε_*i*_(*V*_p_) depends linearly on external strain, that is, voltage *V*_p_ of the piezo drive. Here and in the following, we use the tilde to distinguish operators such as the Pauli-matrices 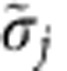 in the eigenbasis from those in the localized basis *σ*_*j*_. The energy splitting in the diagonal basis is 
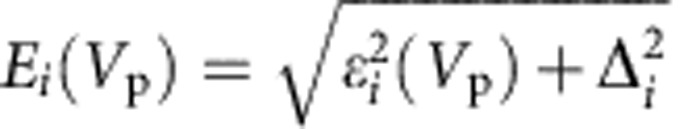
. The transformation to the diagonal basis corresponds to a rotation about the angle *ξ*_*i*_(*V*_p_), defined by tan *ξ*_*i*_=Δ_*i*_/*ε*_*i*_ with sin *ξ*_*i*_=Δ_*i*_/*E*_*i*_ and cos *ξ*_*i*_=*ε*_*i*_/*E*_*i*_. For example, the operators *σ*_z,i_, whose eigenvalues identify the particle positions, transform as


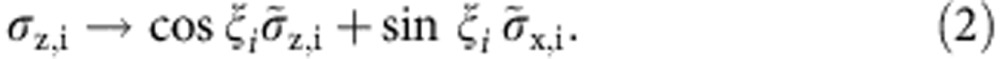


We write the Hamiltionian of two coupled TLS as *H*_T_=*H*_1_+*H*_2_+*H*_12_, with the interaction term,


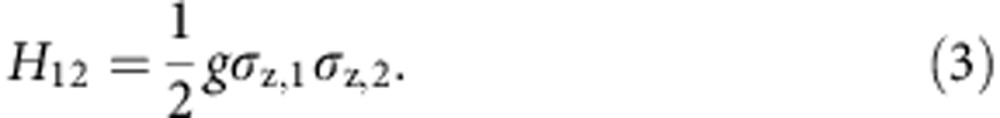


Within the tunnelling model, the defect’s mutual coupling parameter *g* comprises their electric dipole interaction as well as a strain-mediated elastic contribution. Our spectroscopic data does not allow us to distinguish between these coupling mechanisms. However, since TLS interact with the qubit only electrically, we can determine the projections of the TLS’s electric dipole moments onto the field in the qubit junction independently. Details of this analysis are included in the Supplementary Note 2.

In the diagonal basis, the interaction mentioned in equation (3) consists of four terms that are combinations of 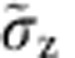 and 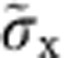 for each TLS (Supplementary Note 3). However, for explaining the observed S-shaped signal (Fig. 3), only two terms are relevant. The energy shift of TLS1, that is, the amplitude of the ‘S’, is given by the longitudinal coupling component 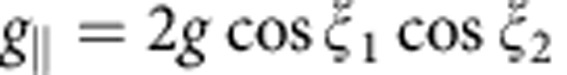 which stems from the term 
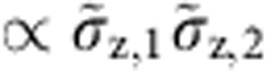
. The transversal component results in exchange coupling 
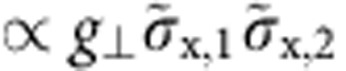
 and defines the size of the level repulsion 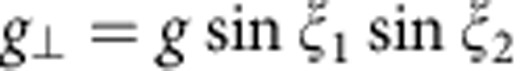. The remaining two parts of 
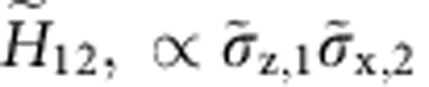
 and 
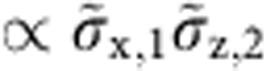
, yield only minor energy shifts and can be neglected to first order. However, in our numerical fits to the spectrum, we take the full interaction equation (3) into account.

### Two-photon swap spectroscopy

**Figure 4:**
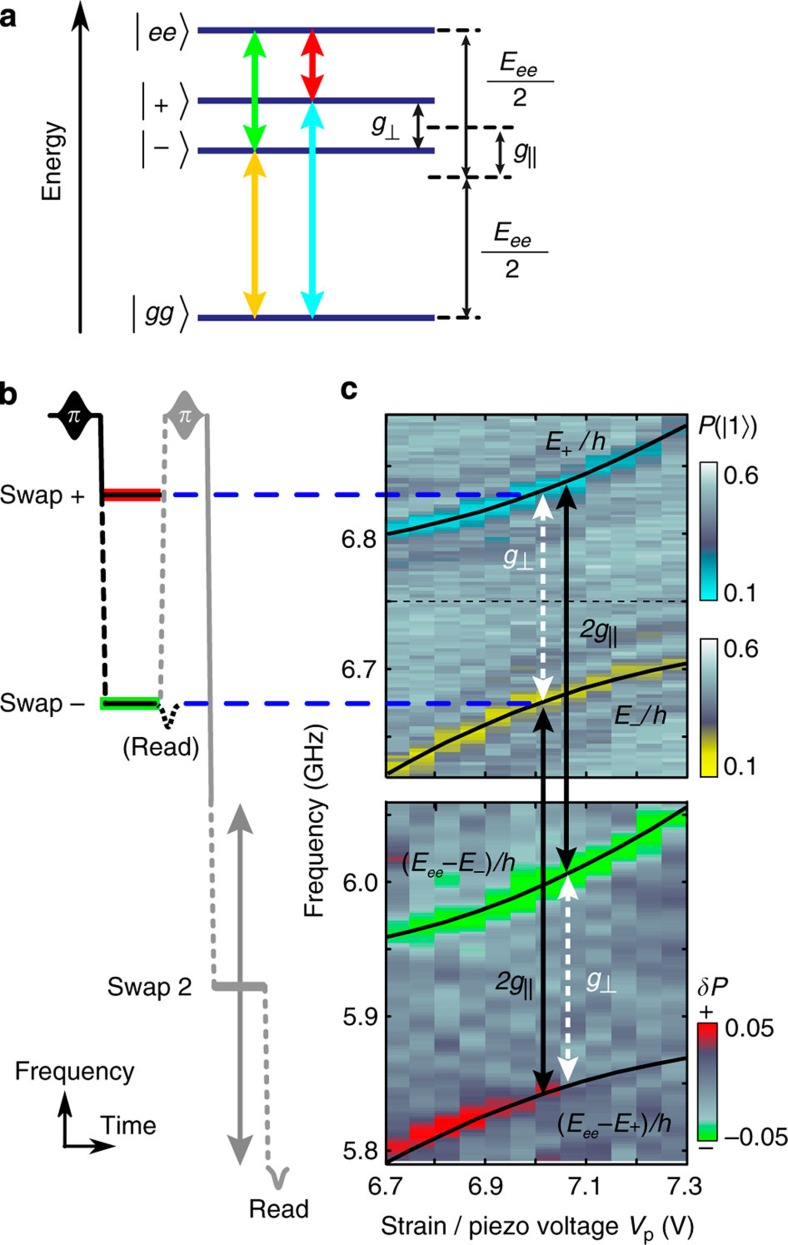
Exploring the energy level structure for the coupled defect system. (**a**) Level scheme of the resonantly coupled defect system for an applied strain near the right avoided level crossing of Fig. 3 at *V*_p_≈7 *V*. Energy splitting and offset of the entangled states 
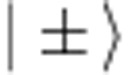
 are determined by transversal and longitudinal coupling strengths 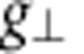 and 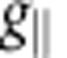, respectively. (**b**) Pulse sequence used to map out the complete energy level structure. For the upper panel in **c**, the qubit was measured after the first swap operation at varying frequencies in order to calibrate the entangled states energies. The lower panel was obtained using the complete sequence: preparation of one of the entangled states 
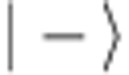
 or 
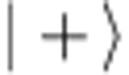
 in a first swap, followed by a second qubit excitation and variation of the second swap frequency to reveal the transitions between the entangled states to the fully excited state 
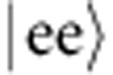
. The colour maps were chosen according to the coloured arrows that indicate the corresponding transitions in **a**. Lines in **c** are calculated from theory.

To fully explore the coherently coupled defect system with even higher precision, we focus on the region near the right anti-crossing where both TLS are strain-tuned into resonance. This results in the four-level energy spectrum illustrated in Fig. 4a, which we map out by performing microwave swap spectroscopy on the system prepared in different entangled states. For this, we follow the sequence sketched in Fig. 4b. The qubit is first prepared in its excited state and then tuned to either the lower or upper branch of the avoided level crossing, realizing a swap operation with the entangled state 
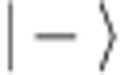
 or 
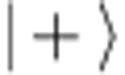
, respectively (Fig. 4a). Directly afterwards, the JJ qubit is again excited and tuned through a lower frequency range in order to find the transition that brings the system of coupled TLS to the fully excited state 
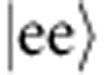
.

Data in the upper panel of Fig. 4c were obtained by measuring the qubit after the first swap operation, thus indicating the avoided level crossing as in our usual defect spectroscopy protocol in order to calibrate the preparation of the chosen entangled state. The complete sequence results in the data shown in the lower panel of Fig. 4c, where we plot the difference in qubit population between two experiments in which the TLS system was prepared in either one of the two entangled state by adjusting the frequency of the first swap pulse. This experiment clearly reveals the transitions to the fully excited state in excellent agreement with theory (solid lines in Fig. 4c). Moreover, we directly obtain the energies *E*_1_ and *E*_2_ of the two unperturbed TLS as well as the longitudinal and transversal coupling strengths as indicated in Fig. 4a. A detailed analysis of this experiment is contained in Supplementary Note 4.

The spectrum calculated from our theoretical model, shown in the inset of Fig. 3, allows one to fit all system parameters and reproduces the data with high accuracy (Supplementary Fig. 3). The inter-TLS coupling strength *g*= −872 MHz is calculated from its components 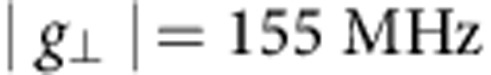 and 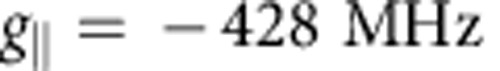, obtained in swap spectroscopy for the TLS tuned into resonance at *V*_p_=7 V as explained above.

Together with the energy splitting *E*_*i*_ of the individual TLS at their resonance, we can determine their strain-dependent mixing angles *ξ*_*i*_ to fully characterize the system. The obtained TLS parameters including their dependence on applied piezo voltage are summarized in Table 1, more details on this evaluation are given in Supplementary Note 3.

A final remark concerns the probability of finding two TLS spaced closely enough to expect an interaction of similar strength as in our experiment. Considering only electric dipole interaction and assuming for both TLS an electric dipole moment of *d*~1 eÅ, which is consistent with the results of our work and agrees with recent observations and theory^4^,^10^,^19^,^20^, we can make a rough estimate of the maximal distance between the two interacting TLS to be on the order of 5 nm (Supplementary Note 4). When distributing the ~50 TLS visible in the accessible frequency range of ~6–9 GHz evenly onto the area of the 1 μm^2^ large qubit junction, on average each TLS occupies an area of ~140 × 140 nm^2^. Thus, although one may expect an increased TLS density at interfaces, observing two coherently interacting TLS in JJs is indeed a rare case.

## Discussion

The experimental techniques presented here provide a novel spectroscopic view onto the bath of sparse material defects by accessing the quantum states of individual TLS and small coupled systems with a superconducting qubit while strain-tuning their internal degree of freedom. This lays the ground for further experiments to clarify the microscopic origin of TLS, which is vital for the advancement of various kinds of nanofabricated devices whose functionality is hampered by defects. So far, the atomic tunnelling model readily explains all effects observed with TLS in tunnel junctions and, in particular, the here demonstrated strong coupling of TLS to mechanical strain and their interaction with both coherent as well as randomly fluctuating defects. Our results open way to detailed testing of the 50-year old tunnelling model on the basis of individual TLS, for example, by performing defect spectroscopy for statistical analyses of the TLS distribution and by studying the strain dependence of TLS coherence.

## Methods

### Superconducting qubit sample

The phase qubit sample^25^ used in this work was fabricated in the group of J. M. Martinis at University of California, Santa Barbara. The qubit junction had an area of about 1 μm^2^, fabricated using aluminium as electrode material and its thermally grown oxide as a tunnel barrier. All data have been obtained at a sample temperature of about 35 mK. The mechanical strain was controlled by bending the sample chip with a piezo transducer^21^.

## Author contributions

The experiments were conceived by J.L., G.J.G., G.W. and A.V.U., and performed by J.L. and G.J.G. Theory and simulations were done by C.M., J.H.C. and G.J.G. The article was written by J.L., G.J.G., G.W. and C.M. with contributions from all authors.

## Additional information

**How to cite this article:** Lisenfeld, J. *et al.* Observation of directly interacting coherent two-level systems in an amorphous material. *Nat. Commun.* 6:6182 doi: 10.1038/ncomms7182 (2015).

## Supplementary Material

Supplementary Information Supplementary Figures 1-4, Supplementary Notes 1-5, and Supplementary References.

## Figures and Tables

**Table 1:** Measured parameters of the two coupled TLS.

	Δ_i_ (GHz)	*ε*_i_(*V*_p_) @ *ε*_2_=0
TLS1	5.47	3.18 GHz to 4 MHz V^−1^
TLS2	1.3	295 MHz V^−1^

TLS, two-level system.

The asymmetry energies *ε*_*i*_ are given at the symmetry point of TLS2 (*V*_p_=−14.05 V).
