# Supplementary material for: Observation of directly interacting coherent two-level systems in an amorphous material
Source: Nat Commun. 2015 Feb 5;6:6182. doi: 10.1038/ncomms7182 (PMC4327544; doi:10.1038/ncomms7182)
Supplement: Supplementary Information — Supplementary Figures 1-4, Supplementary Notes 1-5, and Supplementary References. [file ncomms7182-s1.pdf]

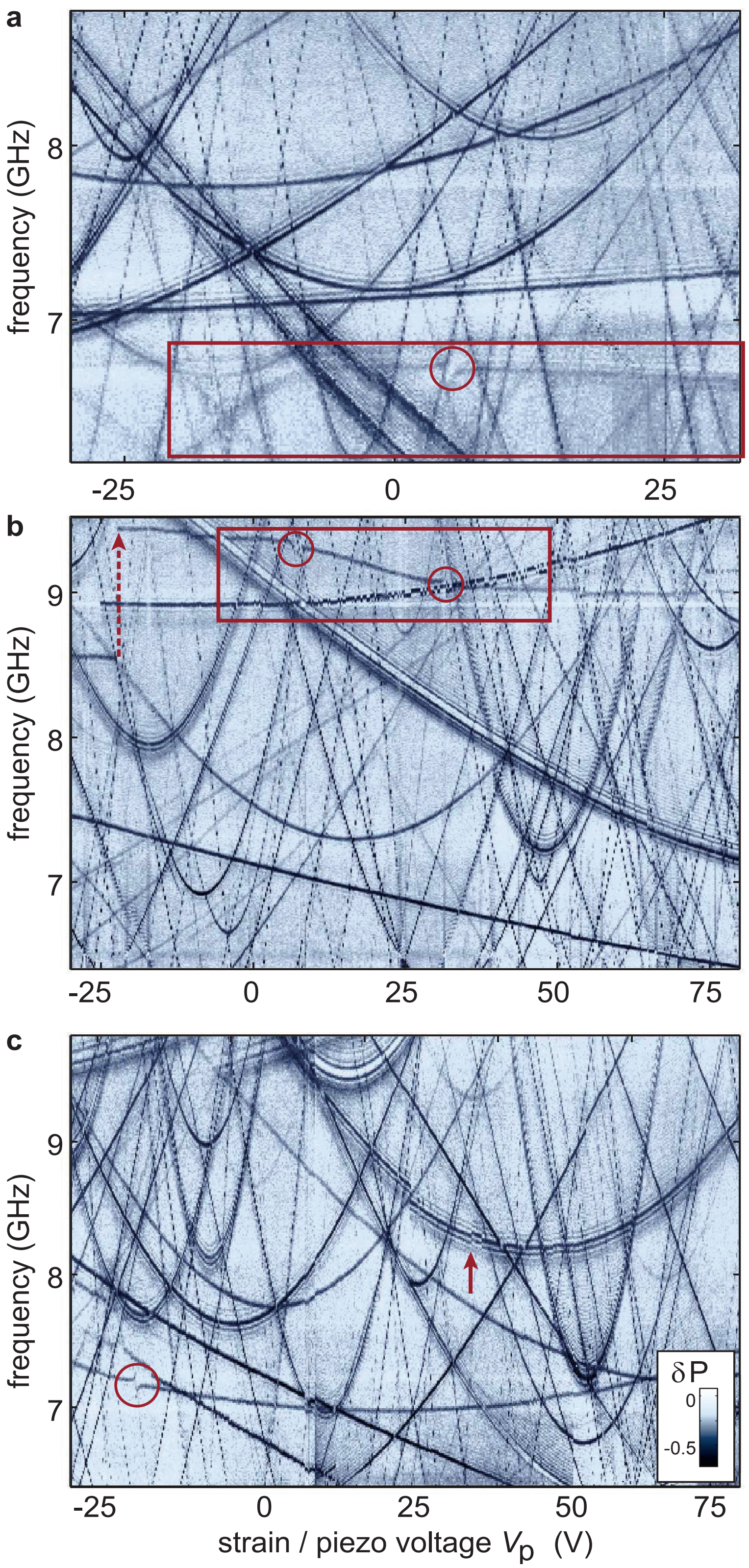

Supplementary Figure 1. Defect spectroscopy examples obtained on the same sample. Red boxes highlight coupled TLS systems, circles indicate avoided level crossings. **a** Data taken in April 2012, showing partly the signature of the coupled TLS system that was characterized in this work. **b** Data acquired soon after cooling down the sample in March 2013, about 3 weeks before taking the data in Fig. 2d of the main manuscript. A few TLS changed their properties, while most remained stable. The dashed arrow indicates a spontaneous change in TLS parameters. **c** Another example of an observed avoided level crossing (circle), and telegraphic switching (arrow).

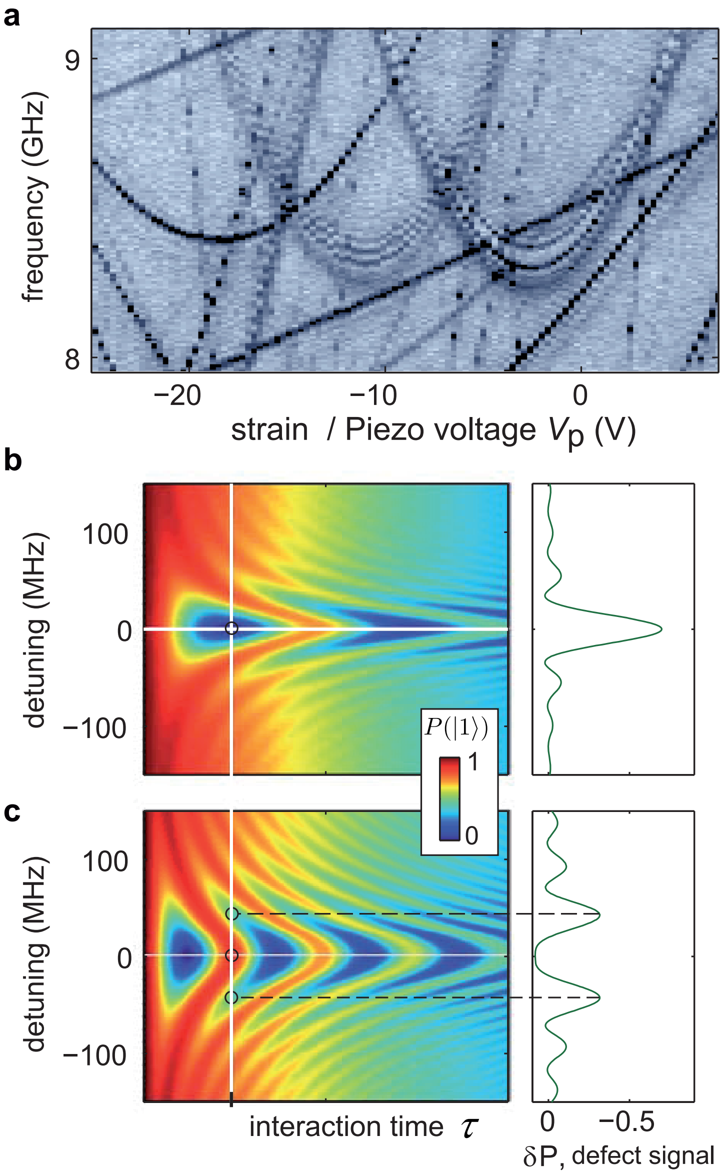

Supplementary Figure 2. **Origin of fringes in defect spectra.a** Defect spectroscopy example, showing the hyperbolic resonance frequency traces of three TLS. Fringes appear around the exact TLS resonance for strongly coupled TLS (middle and rightmost hyperbola). **b** and **c** Calculated population probability $P(|1\rangle)$ of an initially excited qubit, showing coherent oscillations due to coupling to a TLS in dependence of the interaction time τ and detuning (left panels). The right panels show the defect signal δP for a fixed interaction time τ (cross-section along vertical white line in the left panel). For **c**, the TLS-qubit coupling strength is twice larger than for **b**, resulting in faster oscillation. For the more strongly coupled TLS, but same interaction time τ, the signal δP reaches minima at non-zero detuning, giving rise to the pronounced fringes or shadow-lines in defect spectroscopy.

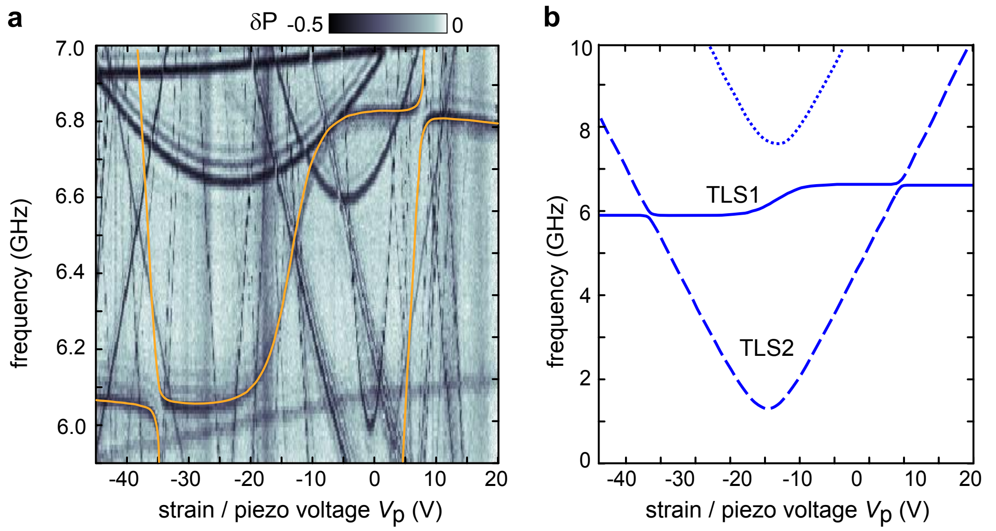

Supplementary Figure 3. **Comparison between experiment and theory.** **a** Strain-spectroscopy data showing the characteristic S-shaped signature of two coupled defects. The calculated spectrum is superimposed on the data, showing the excellent agreement with theory. **b** A plot of the whole theoretical spectrum using the values from Tab. I. The dashed hyperbola visualizes the TLS2 trace invisible in spectroscopy, while the dotted curve shows the energies of the fully excited system.

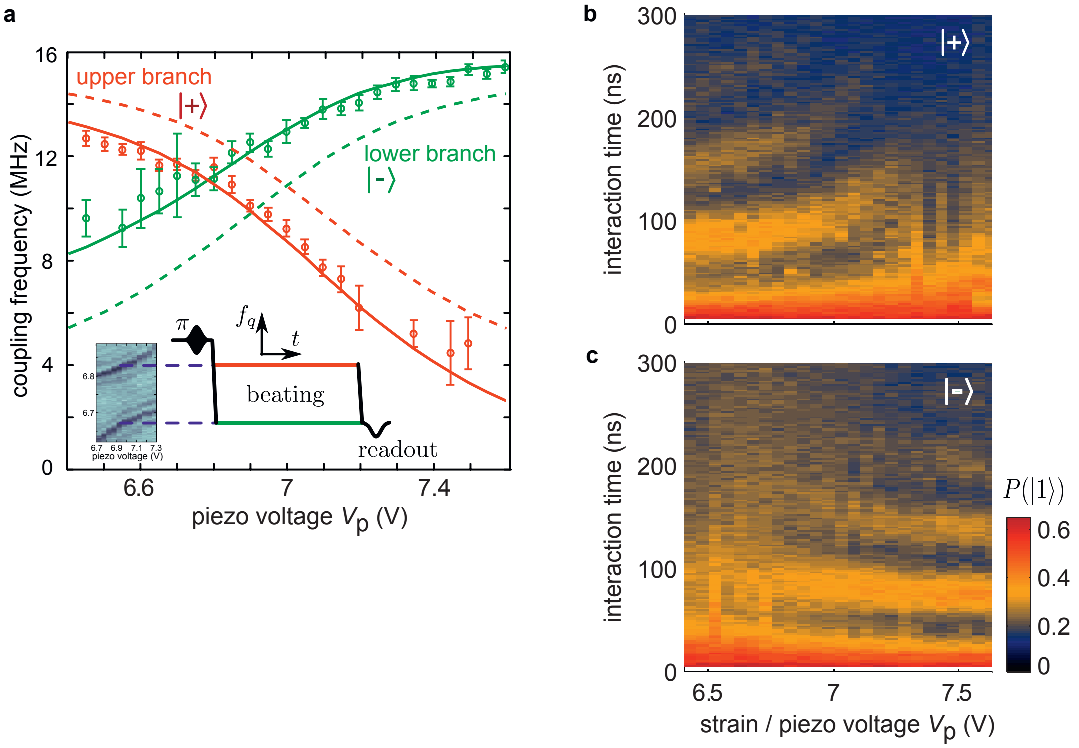

Supplementary Figure 4. **Measuring the coupling strength between the qubit and each TLS.** **a** Measured frequencies of oscillations in the qubit population for the excited qubit tuned to lower and upper branches of the avoided level crossing, respectively, which equals the coupling strength to the corresponding entangled states $|+\rangle$ and $|-\rangle$. The inset shows the used pulse sequence. It can be clearly seen that the measured coupling strengths (circles) deviate from the dashed curves which are calculated for the case of $v_{2,\perp} = 0$. However, one can account for the shift by introducing a non-zero qubit-TLS2 coupling (solid lines). **b** and **c**: Observed coherent swap oscillations in $P(|1\rangle)$ (colour-coded) vs. strain and the interaction time. Their decay is dominated by qubit decoherence.

SUPPLEMENTARY NOTE 1: DEFECT SPECTROSCOPY

Our defect spectroscopy protocol relies on detecting the transition of a single microwave photon, initially stored in the qubit, via resonant interaction to individual TLS. In comparison to our previous method [1], where a long microwave pulse was saturating the qubit transition, here we use a π-pulse to maximize the qubit population probability and thus increases the signal-to-noise ratio. In principle, this method equally detects strongly coupled circuit or environmental resonances, which can however be distinguished by their independence on strain. We did not observe any strain dependence of the qubit parameters nor presumed circuit resonances. We note that a similar, independently developed technique of TLS swap spectroscopy was used in Ref. [2] to obtain statistics on TLS coherence and coupling strength, but without mechanical strain control.

Observation of coupled TLS systems

We performed defect spectroscopy on the same sample and setup in 12 different cool-downs over a course of two years. While the qubit parameters remained constant, the distribution of TLS resonances changed completely. This is not surprising given the observed strong coupling of TLS to the local mechanical strain, which is expected to vary between cool-downs due to small reconfigurations of atomic positions. However, changes in defect spectroscopy can also be explained by offsets in the externally applied strain due to thermal dilatation affecting the sample holder.

S-shaped signatures of coherently coupled defects, shown in Supplementary Figure **1**, were observed in two different cool-downs. Supplementary Figure **1a** was obtained in the experimental run in which we characterized the coupled defect system as discussed in the main text. About one year later, the same sample revealed a similar system of two coupled defects shown in the red box in **1b**, having comparable longitudinal coupling strength but different resonance frequencies and opposite symmetry. It may well be that both observations involve the same tunneling systems which however experience slightly different local potential configurations.

In Supplementary Figure **1b**, one can notice that the frequency of the coupled defect system switched while the spectrum was measured around a voltage of about -25 V (the dashed red arrow indicates the shift in frequency). Such spontaneous changes in the resonance frequencies of some TLS are observed frequently and occur most often shortly after the sample was cooled to milikelvin temperatures. This is readily explained by a coupling of the observed coherent TLS to incoherent TLS, also called "two-level fluctuators", which may be of same physical origin but have a small rate of tunneling between their potential wells. If such a fluctuator is strongly asymmetric, it may be found in its higher energy state soon after the cool-down while it would remain in its ground state once tunneling occurred. Experimentally, we found that the number of spontaneously switching TLS is reduced by repeatedly cycling the applied strain through the complete range, hereby "annealing" the sample in the sense that fluctuators are stimulated to tunnel to their more stable ground state. The data shown in Fig. **2 d** of the main text has been measured in the same cool-down as **1 b**, but 3 weeks later. One can see that some TLS changed their properties in between measurements, while others remained stable. More examples of mutual defect coupling are seen in Supplementary Figure **1c**, showing telegraphic switching of a TLS' resonance frequency (arrow) and an avoided level crossing (circle).

Effects and experimental artefacts

In the following, we discuss some artefacts that may be observed with this spectroscopy technique.

Fringes or shadow-like replica of some TLS traces, such as shown in Supplementary Figure **2a**, may appear for TLS whose coupling strength to the qubit exceeds $h/2\tau$. In this case, the chosen interaction time τ exceeds the duration of one swap operation such that the excitation is transferred back to the qubit, and this lowers the defect signal δP (see also Fig. 2b in main article). Since the frequency of swap oscillations increases with the detuning between qubit and TLS, a minimal qubit population (maximal signal $|\delta P|$) thus occurs only in the vicinity of the exact resonance as shown in Supplementary Figures **2b** and **c**.

Fringes may also occur due to residual entanglement between the qubit and TLS. An example is visible in Supplementary Figure **1c** in a range of $V_\mathrm{p} \approx 40...50$ V at low frequencies. In this measurement, the qubit was biased at a frequency of 6.4 GHz, so that already during excitation it was near resonance with a strongly coupled TLS. Accordingly, the π-pulse prepares some entangled state between the systems, whose phase is then modified in dependence of the subsequent qubit detuning during the interaction time τ.

Qubit drift due to uncontrolled slow changes in its bias flux leads to deviations in the defect signal's frequency dependence. For Supplementary Figure **1c**, in total about 140,000 data points were measured, of which each was averaged 1000 times at a repetition rate of about 500 Hz, resulting in a total measurement duration of $\approx$ 3.5 days. An example of qubit drift can be seen in Supplementary Figure **1c** around a piezo voltage of about 7 V, which was measured while feeding liquid Helium to the dilution refrigerator. This resulted in a small change of the qubit flux bias because of temperature variations in its bias line filters that are installed at the 1K-pot of the cryostat. Qubit drift can be distinguished from TLS resonance frequency fluctuations since all TLS signals are shifted equally. We note that the measurement time could be easily reduced by at least one order of magnitude if one employs a dispersive

qubit readout method [3–5] instead of the DC-SQUID switching-current measurements employed here [6].

SUPPLEMENTARY NOTE 2: THE QUBIT-TLS COUPLING

Assuming the TLSs interact with their environment via their electric dipole moment with the qubit circuit, the corresponding coupling operator is σ_z, whose expectation values identify the position of the particle. The interaction of an individual TLS with the electric fields inside the qubit's junction is then described as

$$H_{iq} = \frac{1}{2} v_i \tilde{\tau}_x \sigma_{z,i} \, , \tag{1}$$

where v_i is the electric dipole interaction strength and we used the fact that for a phase qubit in its eigenbasis, the electric field operator is $\propto \tilde{\tau}_x$ [7]. Rewriting Supplementary Eq. (1) in the TLS eigenbasis, we retain two terms, of which the transversal coupling term $v_{i,\perp}\tilde{\tau}_x\tilde{\sigma}_{x,i}$ with $v_{i,\perp} = v_i \sin\xi_i$ describes the resonant interaction between the JJ qubit and the TLS i. It is this term in particular which causes the exchange of excitations between the two systems.

We obtained the electric coupling strength between qubit and TLS1, $v_{1,\perp}$, by fitting time-domain oscillations of the initially excited qubit tuned into resonance with TLS1, far away from the TLS1-TLS2 anti-crossing, where $v_{1,\perp}$ is independent of the state of TLS2. We performed the same experiment also with both, lower and upper branches, in the vicinity of the TLS1-TLS2 anti-crossing (the pulse sequence is shown on the inset in Supplementary Figure **4**). The result is plotted in Supplementary Figure **4a**, green for the lower and red for the upper branch. The panels in Supplementary Figure **4**b and c show the observed oscillations in the qubit population.

Since TLS2 is very weakly coupled to the qubit, the oscillation frequency decreases quickly with voltage when following a branch turning into TLS2. However, it is worth noting that the crossing point of the two curves is not at the voltage of 7 V where TLS1 and TLS2 are in exact resonance. This would be expected if the qubit-TLS2 coupling was zero, as indicated by the dashed curves. A non-zero $v_{2,\perp}$ explains this shift, because the coupling between the qubit and the $|\pm\rangle$ states scales as $(v_{1,\perp} \pm v_{2,\perp})/\sqrt{2}$. Then, the theory shows a very good agreement with the experiment and we obtain $v_{1,\perp} = 15.4$ MHz and $v_{2,\perp} = 3.0$ MHz at an applied strain where the TLS are in resonance $E_1 = E_2$.

With the already known ξ_1 and ξ_2, $v_1 = 18.7$ MHz and $v_2 = 14$ MHz can be calculated from Supplementary Equation (4). It is worth mentioning that the very small $v_{2,\perp}$ is not attributed to a smaller dipole moment in direction of the qubit electric field, but can be simply explained by a strong asymmetry $\Delta_2 \ll \varepsilon_i$ resulting in a very small dipole moment of the TLS eigenstates. This analysis is restricted to the assumption that both TLSs interact purely electrically.

SUPPLEMENTARY NOTE 3 : ANALYSIS OF THE FULL HAMILTONIAN

We write the full Hamiltonian describing the JJ-qubit coupled to two interacting TLSs as

$$H = H_q + \sum_{i=1}^{2} H_i + \sum_{i=1}^{2} H_{iq} + H_{12} \, , \tag{2}$$

with the qubit Hamiltonian $H_q = \frac{1}{2}E_q\tilde{\tau}_z$, where E_q is the level-splitting of the two lowest qubit states and $\tilde{\tau}_z$ is a Pauli-matrix describing the qubit eigenstates. H_i is the Hamiltonian of the ith TLS, while H_{iq} describes its interaction with the qubit, and H_{12} the TLSs' mutual interaction as described in the main manuscript.

Diagonalizing the Hamiltonian in Supplementary Eq. (2) introduces several new terms. The resulting Hamiltonian, $\widetilde{H}$, can be significantly simplified by ignoring all coupling terms of the form $\propto \sigma_x\sigma_z$ and $\propto \sigma_z\sigma_x$. They represent a coupling where one partner changes its state depending on the instantaneous state of the other subsystem and contribute only as small energy offsets. Neglecting these minor energy shifts, we can write the significant parts of the full Hamiltonian as

$$\begin{aligned} \widetilde{H} = \frac{1}{2}(&E_q\tilde{\tau}_z + E_1\tilde{\sigma}_{z,1} + E_2\tilde{\sigma}_{z,2} + g_{\parallel}\tilde{\sigma}_{z,1}\tilde{\sigma}_{z,2} \\ &+ g_{\perp}\tilde{\sigma}_{x,1}\tilde{\sigma}_{x,2} + v_{1,\perp}\tilde{\sigma}_{x,1}\tilde{\tau}_x + v_{2,\perp}\tilde{\sigma}_{x,2}\tilde{\tau}_x), \end{aligned} \tag{3}$$

with

$$
\begin{aligned}
E_i &= \sqrt{\varepsilon_i^2 + \Delta_i^2}\,, \\
v_{1,\perp} &= v_1 \sin\xi_1 \quad , \quad v_{2,\perp} = v_2 \sin\xi_2\,, \\
g_{\parallel} &= g\cos\xi_1\cos\xi_2 \quad , \quad g_{\perp} = g\sin\xi_1\sin\xi_2\,, \\
\cos\xi_i &= \varepsilon_i/E_i \quad , \quad \sin\xi_i = \Delta_i/E_i
\end{aligned} \tag{4}
$$

Here the relationship between the longitudinal and transversal coupling factors $g_{\parallel}$ and $g_{\perp}$, which are easily identifiable in experiment, and the TLS mixing angles ξ_i becomes clear. In our case, the ξ_i dependence of $g_{\perp}$ cannot be observed because when TLS2 is detuned from TLS1, the perpendicular coupling $\propto \tilde{\sigma}_{x,1}\tilde{\sigma}_{x,2}$ becomes irrelevant We can interpret the term $\cos\xi_i$ as representing the expectation value of the position operator in the double-well potential $\langle\sigma_{z,i}\rangle$, so that $g_{\parallel} = g\,\langle\sigma_{z,1}\rangle\,\langle\sigma_{z,2}\rangle$. As explained in the main manuscript, when tuning through the TLS2 symmetry point, $\langle\sigma_{z,2}\rangle$ changes its sign, corresponding to reversing the direction of TLS2's dipole moment. Due to the strong dipole-dipole interaction, TLS1 adjusts its energy splitting accordingly, and this results in the observed S-shaped signature.

Additionally, the angles ξ_i also change the qubit-TLS coupling strength $v_{i,\perp}$ which determines the visibility of TLS in our defect spectroscopy. The coupling strength $v_{i,\perp}$ is maximal when the TLS is at its symmetry point $\varepsilon_i = 0$, and it is strongly suppressed for largely asymmetric TLSs with $\Delta_i \ll \varepsilon_i$. These considerations allow us to obtain a close fit of the theoretical model to the experimental data, with the results displayed in Supplementary Figure **3** and table I, respectively.

During our measurements, we occasionally observe abrupt jumps of individual TLS' resonance frequencies on a time-scale of days or weeks. From defect spectroscopic data obtained before and after such events, we found that the defects experience changes of both their asymmetry and tunneling energies, while the strain dependence of ε remains very similar. A sudden change in TLS parameters also occurred between measurements of the defect spectrum and that of the fully excited TLS1-TLS2 system (see Appendix B), by which the TLS1 energy shifted by approximately 100 MHz towards lower frequencies. Since after the jump, the theoretical spectrum is found to still coincide with the anti-crossing on the left side of the "S" (data not shown), we can conclude that Δ_2 did not change appreciable in comparison to ε_2 at the TLS1-TLS2 anti-crossing.

SUPPLEMENTARY NOTE 4: MEASURING THE ENERGY OF THE FULLY EXCITED TLS1-TLS2 SYSTEM

The aim of the experiment discussed here is to measure the transition energies of the coupled TLS1-TLS2 system around the anti-crossing at $V_{\rm p} = 7$ V on the right side of the "S"-shaped signature shown in Supplementary Figure **3**. The energies of the four levels $|gg\rangle$, $|\pm\rangle$ and $|{\rm ee}\rangle$ are illustrated in Fig. 4 a. While the splitting size equals $g_{\perp}$, the energetic shift of the anti-crossing from $E_{\rm ee}/2$, which corresponds to the unperturbed TLS energies E_1 and E_2, yields $g_{\parallel}$ [7, 8]. Fig. 4 b visualizes the pulse sequence for this experiment. The upper image of Fig. 4 c shows a zoom of the right anti-crossing with the two branches. After exciting the qubit with a π-pulse, the excitation is swapped to one of the branches by tuning the qubit for the swap time to the corresponding energies around E_+ or E_-. Afterwards, the qubit is excited a second time and tuned to lower frequencies in order to search for the transition that fully excites the TLS system. This occurs at energies around $E_{\rm ee} - E_+$ or $E_{\rm ee} - E_-$, respectively, and the qubit will loose its excitation yielding an additional dark trace in the spectrum image (data not shown). The two plots obtained in this way, one for each branch into which the first excitation was swapped, are subtracted from each other yielding peaks or dips at the relevant energies and zero for the background (Fig. 4 d, lower plot). For better visibility, a color-map has been chosen such that peaks in Fig. 4 c, which correspond to first exciting the lower branch, appear in green and the dips, arising if the qubit excitation was first swapped to the upper branch, show up red. The coupling constants between the two TLSs, and also the TLS energies in the uncoupled case, can now be directly extracted from the data:

$$
\begin{aligned}
2E_1 = 2E_2 &= E_+ + (E_{\rm ee} - E_+) = E_- + (E_{\rm ee} - E_-) \\
g_{\perp} &= E_+ - E_- = (E_{\rm ee} - E_-) - (E_{\rm ee} - E_+) \\
2g_{\parallel} &= E_+ - (E_{\rm ee} - E_-) = E_- - (E_{\rm ee} - E_+).
\end{aligned} \tag{5}
$$

With the known ratio of the tunneling and asymmetry energies of TLS2, ξ_2, g and ξ_1 (Supplementary Eq. (4)) can be calculated (see Tab. I).

SUPPLEMENTARY NOTE 5: DISTANCE BETWEEN THE TLS

In order to obtain a rough estimate of the distance between the coupled TLS, several assumptions have to be made. First of all, for the calculation we assume that the mutual TLS interaction is solely due to electrical dipole coupling, i.e. we neglect any contribution due to elastic coupling. The interaction strength can then be written as [9]

$$\frac{g}{2} = \frac{1}{4\pi\varepsilon_0\varepsilon_r r^3}\left(\vec{\mu}_{1\perp}\vec{\mu}_{2\perp} - 2\vec{\mu}_{1\parallel}\vec{\mu}_{2\parallel}\right), \tag{6}$$

where $\vec{r}$ is the relative position vector from TLS1 to TLS2 and their electrical dipole moments $\vec{\mu}_i = \vec{\mu}_{i,\parallel} + \vec{\mu}_{i,\perp}$ are decomposed into components parallel and perpendicular to $\vec{r}$, respectively.

We estimate the magnitude of the TLS' electrical dipole moments by their components parallel to the electric field in the Josephson junction, which can be determined as described in Supplementary Note 3. The qubit-TLS coupling strength $v_{i,\parallel}$ is the product between the TLS dipole moment and the electrical field in the junction [10],

$$v_{i,\parallel} = 2\frac{\mu_{i,\parallel}}{x}\sqrt{\frac{E_{10}}{2C}}, \tag{7}$$

where $x \approx 2$ nm is the thickness of the Junction's tunnel barrier, $C = 850$ fF the qubit's capacitance and $E_{01} \approx h \cdot 6.5$ GHz the qubit's energy level splitting at resonance with the TLS. Using the values of Table I, we obtain $\mu_{1,\parallel} \approx 0.46$ eÅ and $\mu_{2,\parallel} \approx 0.38$ eÅ. Finally, we assume $\mu_i = \mu_{i,\parallel}$ and use Supplementary Eq. (6) with $\epsilon_r = 10$ (saphhire) to estimate the maximal distance between the TLS, which results in r = 4.1 nm and r = 5.2 nm, for horizontal and vertical arrangements of the TLSs, respectively. We note that this maximal vertical distance exceeds the thickness of the tunnel barrier.

SUPPLEMENTARY REFERENCES

[1] Grabovskij, G. J., Peichl, T., Lisenfeld, J., Weiss, G. & Ustinov, A. V. Strain Tuning of Individual Atomic Tunneling Systems Detected by a Superconducting Qubit. *Science* **338**, 232–234(2012).

[2] Shalibo, Y. *et al.* Lifetime and Coherence of Two-Level Defects in a Josephson Junction. *Phys. Rev. Lett.* **105**, 177001–177004 (2010).

[3] Wirth, T., Lisenfeld, J., Lukashenko, A. & Ustinov, A. V. Microwave readout scheme for a Josephson phase qubit. *Applied Physics Letters* **97**, 262508 (2010).

[4] Jerger, M. *et al.* Readout of a qubit array via a single transmission line. *EPL (Europhysics Letters)* **96**, 40012 (2011).

[5] Chen, Y. *et al.* Multiplexed dispersive readout of superconducting phase qubits. *Applied Physics Letters* **101**, 182601 (2012).

[6] Wallraff, A. *et al.* Switching current measurements of large area josephson tunnel junctions. *Review of Scientific Instruments* **74**, 3740-3748 (2003).

[7] Cole, J. H. *et al.* Quantitative evaluation of defect-models in superconducting phase qubits. *Appl. Phys. Lett.* **97**, 252501 (2010).

[8] Lupascu, A., Bertet, P., Driessen, E. F. C., Harmans, C. J. P. M. & Mooij, J. E. One- and two-photon spectroscopy of a flux qubit coupled to a microscopic defect. *Phys. Rev. B* **80**, 172506–172509 (2008).

[9] Kocbach, L. & Lubbad, S. Geometrical simplification of the dipole-dipole interaction formula. *Physics Education* **45**, 345 (2010).

[10] Martinis, J. M. *et al.* Decoherence in Josephson Qubits from Dielectric Loss. *Phys. Rev. Lett.* **95**, 210503–210506 (2005).
